# Control over the Self-Assembly of Supramolecular Hydrogels Using Hofmeister Effect

**DOI:** 10.3390/gels12080693

**Published:** 2026-08-04

**Authors:** Lai Wei, Qi Gao, Hongwang Tang, Xuhong Guo, Yiming Wang

**Affiliations:** 1 School of Chemical Engineering, East China University of Science and Technology, Shanghai 200237, China; 2 Shanghai Key Laboratory for Intelligent Sensing and Detection Technology, Institute of Intelligent Sensing and Instruments, East China University of Science and Technology, Shanghai 200237, China

**Keywords:** Hofmeister effect, self-assembly, hydrogels, low-molecular-weight gelators, supramolecular chemistry

## Abstract

Supramolecular hydrogels are usually prepared in aqueous media containing diverse ions, for instance, buffer solutions, yet the influences of ions, especially the Hofmeister effect, on their self-assembly are often overlooked. Here, we systematically investigate the impacts of different anions in the Hofmeister series on the self-assembly of supramolecular hydrogels. On the basis of a hydrazone formation-mediated supramolecular gelation system, we found that the addition of kosmotropic anions can accelerate the self-assembly of gelators as determined by rheology and critical gelation concentration tests. Confocal microscopy observations and rheological measurements demonstrate that the resultant hydrogels have denser fibrous networks and higher stiffness relative to the samples without additional kosmotropic anions. In contrast, chaotropic anions effectively impede the self-assembly process. These impacts of ions on the self-assembly of supramolecular hydrogels are in line with the specific ion effect. This work suggests that the Hofmeister effect can serve as an effective approach to control the self-assembly and the properties of supramolecular hydrogels, and the effects of ions should be considered in the studies of supramolecular hydrogels.

## 1. Introduction

Supramolecular hydrogels self-assembled from low-molecular-weight gelators *via* non-covalent interactions show reversible stimulus-responsiveness and tunable mechanical properties [1,2,3,4,5], leading to unique advantages in drug delivery [6,7,8,9], tissue engineering [10,11,12,13], and lubricating [14,15,16,17]. Currently, various chemical and physical stimuli have been explored to control the self-assembly process and properties of supramolecular hydrogels [18,19,20,21,22]. The vast majority of supramolecular hydrogels are typically prepared in aqueous solutions that contain a variety of ionic species, for instance, buffer solutions [23,24,25,26]. It is well-known that the ions can influence the screening of electrostatic interactions, thereby affecting the self-assembly of supramolecular hydrogels that are associated with charges [27,28,29,30]. However, the Hofmeister effect, as a powerful way to regulate intermolecular interactions, is rarely used to control the self-assembly of supramolecular hydrogels [31,32].

In 1888, Frank Hofmeister proposed the Hofmeister effect, which, for the first time, systematically elucidated the differential regulatory mechanisms of different inorganic salt ions on the stability and solubility of biomacromolecules in aqueous systems from a physicochemical perspective [33]. He categorized ions based on their relative ability to either decrease (kosmotropes) or increase (chaotropes) protein solubility, thereby establishing the Hofmeister ion series, which has been a fundamental theoretical framework [34,35,36]. Through its modulation of hydration, the Hofmeister effect provides a new approach for controlling molecular self-assembly and for exploiting ion-specific regulation of soft materials [37,38,39]. Recent studies have revealed that the Hofmeister effect can not only modulate the mechanical properties of polymer hydrogels and influence enzymatic catalytic efficiency but also serve as an effective strategy for the controllable construction of supramolecular assembly pathways [40,41,42,43], enabling the preparation of smart ion-responsive supramolecular hydrogels [44,45,46]. For example, Roy et al. demonstrated that different Hofmeister ions can markedly influence the self-assembly of peptide amphiphiles by modulating hydrophobic interactions [47]. Beyond its historical context in protein chemistry, the Hofmeister effect has increasingly been recognized as a versatile physicochemical principle for engineering ion-responsive soft materials, particularly in directing the self-assembly of supramolecular systems through specific ion–solute and ion–water interactions.

Recently, our group has reported dynamic self-assembly of supramolecular hydrogels by orchestrating the Hofmeister effect through a simple enzymatic reaction [48]. Despite these advances, investigations of the influences of specific ions on the self-assembly of supramolecular hydrogels remain scarce.

**Figure 1:**
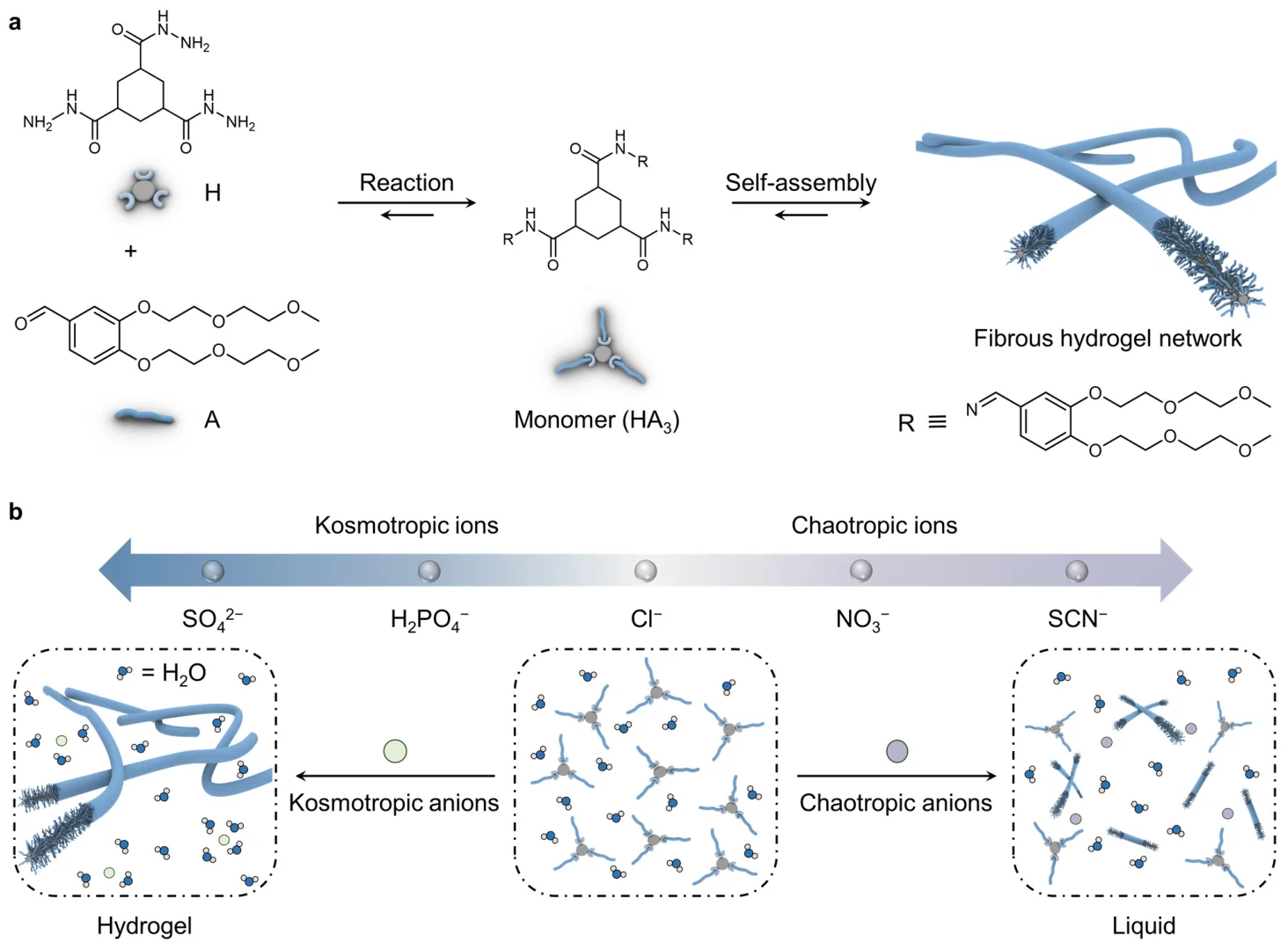
Scheme showing the self-assembly of hydrazone-based supramolecular hydrogel controlled by the Hofmeister effect. (**a**) The supramolecular gelation system in which the monomer HA_3_ is formed from the building blocks of H and A and self-assembles into a fibrous hydrogel network; (**b**) illustration showing the Hofmeister series and the influences of different anions on the self-assembly of HA_3_.

In this work, relying on a hydrazone formation-mediated supramolecular gelation system, we demonstrate that the Hofmeister effect can significantly influence the self-assembly and the material properties of supramolecular hydrogels. In this system, the hydrazone-based gelator (HA_3_) is formed from non-assembling tris-hydrazide (H) and aldehyde (A) via hydrazone formation. When the concentration of the formed HA_3_ exceeds the critical aggregation concentration (CAC), they will self-assemble into fibers, which entangle together to form supramolecular hydrogels (Figure 1a) [19,49,50,51]. Five anions (SO_4_^2−^, H_2_PO_4_^−^, Cl^−^, NO_3_^−^, SCN^−^) spanning the Hofmeister series are used to test their effects on the gelation process (Figure 1b). We find that kosmotropic anions (SO_4_^2−^, H_2_PO_4_^−^) can effectively accelerate the gelation, resulting in hydrogels with denser fibrous networks and higher stiffness (Figure 1b). In contrast, chaotropic anions (Cl^−^, NO_3_^−^, SCN^−^) show an obvious inhibition effect on the self-assembly of the gelators. Importantly, our findings are consistent with the Hofmeister series, indicating the important role of the Hofmeister effect in the study of supramolecular hydrogels.

## 2. Results and Discussion

To investigate the influences of the Hofmeister effect on the self-assembly of supramolecular hydrogels, we utilized a hydrazone formation-mediated supramolecular gelation system that has been investigated in previous work (Figure 1a) [49]. The gelator HA_3_ is in situ formed from tris-hydrazide (H) and aldehyde (A) through the formation of hydrazone bonds, which subsequently self-assemble into a fibrous gel network. The synthesis of these gelation precursors can be found in Supporting Information (Figures S1–S3). Unless stated otherwise, the prepared gelation samples contain 20 mM H and 120 mM A in 10 mM phosphate buffer at pH 6.0. The 20 mM gelator concentration was selected to ensure robust gelation, and the low buffer concentration (10 mM) was chosen to minimize the effect of salt from the buffer on the self-assembly process [19,52]. To investigate the effects of anions on the self-assembly of hydrogel, SO_4_^2−^, H_2_PO_4_^−^, Cl^−^, NO_3_^−^, and SCN^−^ spanning the Hofmeister series were introduced into the gelation samples at prescribed concentrations. Na^+^ was chosen as the counterion for all salt conditions to specifically investigate the anion effects, as Na^+^ is known to exhibit ignorable Hofmeister effects [34,35,36,37]. The ratio of [H]:[A] was kept at 1:6 to ensure a complete conversion of H into HA_3_ according to a previous study.

**Figure 2:**
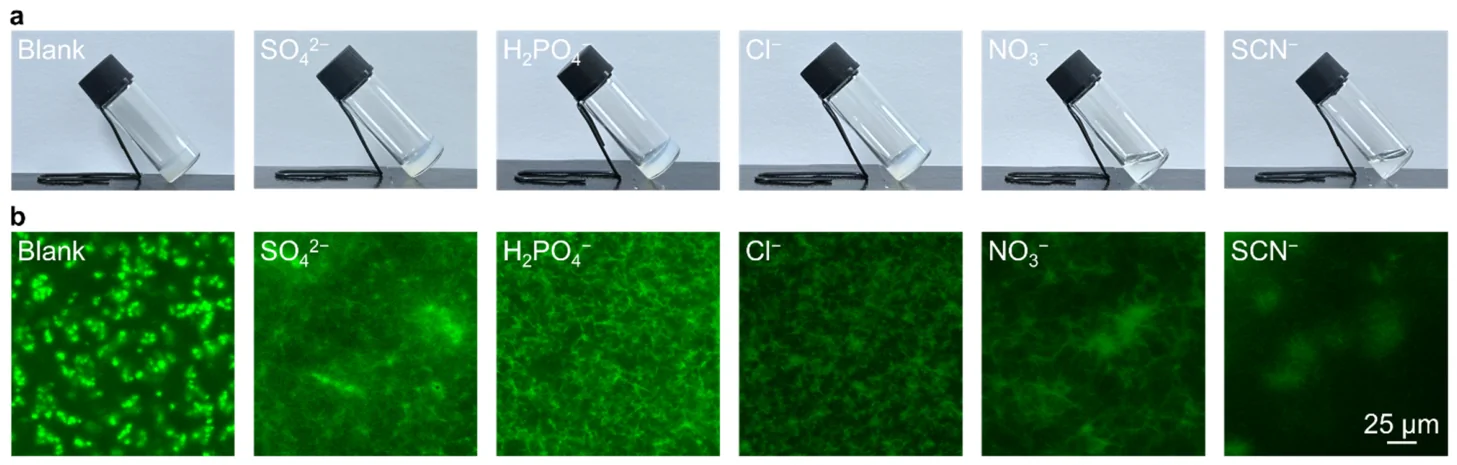
Gelation behaviors of the samples with different anions. (**a**) Photographs showing the gelation results of the samples with different anions, [anion] = 300 mM in each anion-containing sample, and (**b**) confocal microscopy images showing the morphologies of the corresponding samples in (**a**).

In a typical experiment, gelation samples containing 300 mM of different anions were prepared and incubated overnight. We found that the samples without the addition of extra anions and that contain SO_4_^2−^ and H_2_PO_4_^−^ generated stable hydrogels (Figure 2a). The hydrogels formed in the presence of SO_4_^2−^ and H_2_PO_4_^−^ are relatively transparent compared with the blank hydrogel formed without additional anions (Figure S4). The sample containing Cl^−^ formed a relatively weak hydrogel. In contrast, in the cases of NO_3_^−^ and SCN^−^, flowable liquids rather than hydrogels are formed (Figure 2a). Notably, the formed hydrogels driven by kosmotropic anions are stable even with the removal of the anions, as the kosmotropic anions enable the self-assembly of the gelators into a more thermodynamically stable state [52].

To gain further insight into the different gelation results, we characterized the microscopic morphologies of the samples using confocal microscopy observation by adding additional 30 μM aldehyde-decorated fluorescein (A-FL) to label the self-assembled structures (Figure S5). As shown in Figure 2b, all the hydrogel samples show coarse fibrous networks. Interestingly, the samples formed with the addition of SO_4_^2−^ and H_2_PO_4_^−^ show a much denser network compared with the blank hydrogel sample. This observation also explains the relatively transparent optical property of the hydrogels formed with SO_4_^2−^ and H_2_PO_4_^−^. The hydrogel sample formed in the presence of Cl^−^ also presented a fibrous network but was relatively sparse with respect to that of SO_4_^2−^ and H_2_PO_4_^−^. In stark contrast, the samples formed with NO_3_^−^ and SCN^−^ manifest large and sparse fibrous clusters, which explains the observed failure of gelation. Previous studies have demonstrated that the addition of kosmotropic ions can reorganize the structure of water to strengthen the intermolecular interactions among the solutes, while the chaotropic anions lead to an increase in the solvation of solutes [35,48]. These observations are consistent with the previously demonstrated Hofmeister effect, i.e., kosmotropic ions accelerate the self-assembly of gelators into hydrogels by strengthening their intermolecular interactions, while chaotropic anions accelerate dissolving of gelators, thereby inhibiting the occurrence of gelation.

**Figure 3:**
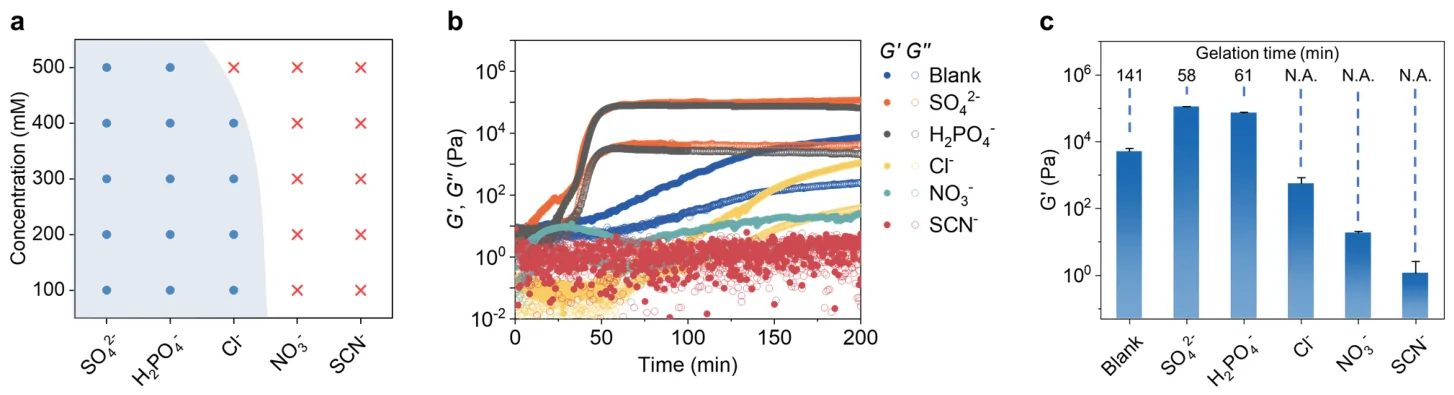
Effects of different anions on the gelation process. (**a**) Dependence of gelation behaviors on the species and concentrations of anions; (**b**) evolution of G′ and G″ of the samples containing 500 mM of different anions over time; and (**c**) plateau G′ and gelation time of the gelation samples extracted from (**b**). N.A. in (**c**) indicates that the samples containing the corresponding anions do not undergo gelation, and therefore, gelation time cannot be confirmed.

Next, we systematically investigated the dependence of gelation behavior on the concentrations of anions (Figure 3a). For kosmotropic anions (SO_4_^2−^ and H_2_PO_4_^−^), hydrogels can be formed across the entire tested concentration range. In the case of Cl^−^, stable hydrogels were obtained only when its concentration was less than 400 mM. For the samples containing chaotropic anions NO_3_^−^ or SCN^−^, gelation cannot occur even after decreasing their concentration to 100 mM.

According to the gelation results discussed above, we further investigated the gelation process of these samples containing different anions using rheology. The concentrations of anions were kept at 500 mM as this concentration clearly identifies the difference between kosmotropic and chaotropic anions in the Hofmeister series. Compared with the control sample without additional anions, the introduction of kosmotropic anions (SO_4_^2−^ and H_2_PO_4_^−^) dramatically accelerated the gelation process and significantly enhanced the mechanical strength of the resulting hydrogels (Figure 3b and Figure S6). Specifically, the maximum storage modulus (G’) of the resultant hydrogels reached 117.4 and 65.2 kPa, respectively, which is significantly higher than that of the blank hydrogel (7.3 kPa). Meanwhile, the gelation time (the time for G’ to reach the plateau) was 58 and 61 min, respectively, which are obviously shorter than that of the blank sample with 10 mM buffer solution (141 min) (Figure 3c). In comparison, the samples containing chaotropic anions show rather low modulus that cannot be considered as a gelation process (Figure 3b).

These results further suggest that kosmotropic anions can promote the self-assembly process, thus leading to fast gelation and stiffer hydrogels, while chaotropic anions show an effective inhibition effect on the gelation process. As such, it is clear that this dependence of gelation results on the species of anions is in line with the Hofmeister effect.

**Figure 4:**
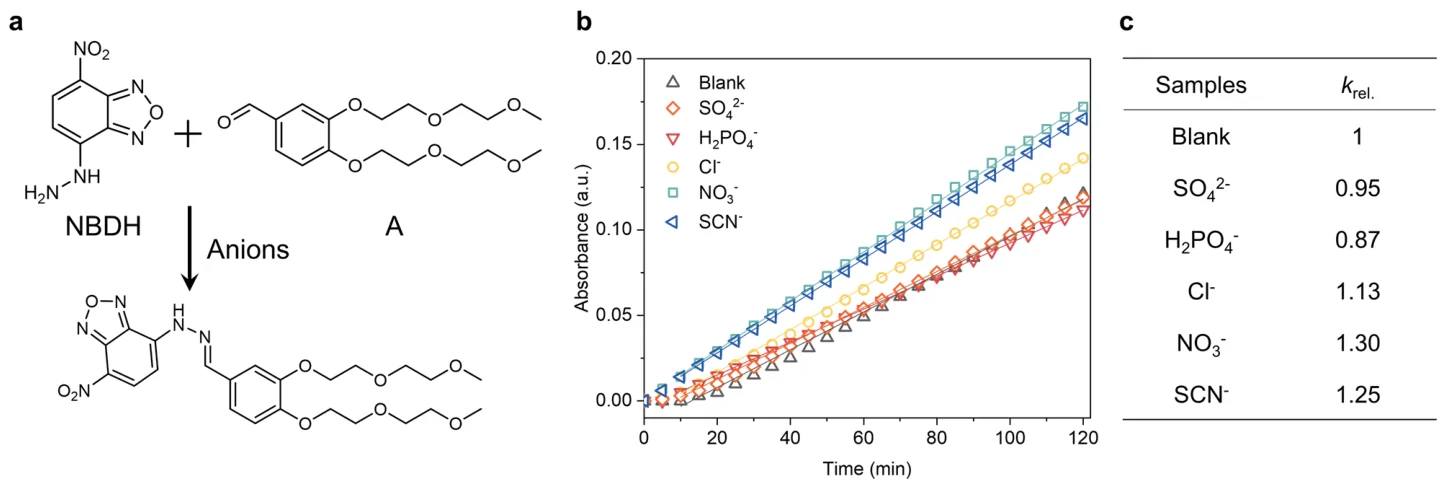
Influence of anions on the formation of the hydrazone bond. (**a**) The model reaction between NBDH and A for kinetic measurement; (**b**) UV-Vis absorbance of the hydrazone product at 480 nm over time at different anions; and (**c**) relative reaction rate constant, *k*_rel_., of the model reaction at different anions. Samples: [NBDH] = 0.045 mM, [A] = 2.5 mM, and [anion] = 100 mM in 10 mM phosphate buffer at pH 6.0.

Next, we are motivated to verify the mechanism of the different gelation behaviors caused by the different anions. Previous studies have demonstrated that the formation rate of HA_3_ can effectively influence the gelation process and properties of the resultant hydrogels [19,49,53]. To rule out this case, we investigated the formation kinetics of the hydrazone bond in the presence of 100 mM anions using a model reaction between nitrobenzoxadiazole hydrazide (NBDH) and aldehyde A (Figure 4a). We found that the relative reaction rates (relative to the group with no additional ions, *k*_rel._) were around 1.0 for all the tested samples (Figure 4b,c), though, somehow, the chaotropic anions show very slight catalysis on the hydrazone formation. As such, the accelerated gelation with kosmotropic anions and the inhibited gelation with chaotropic anions do not originate from the changes in the kinetics of hydrazone formation.

**Figure 5:**
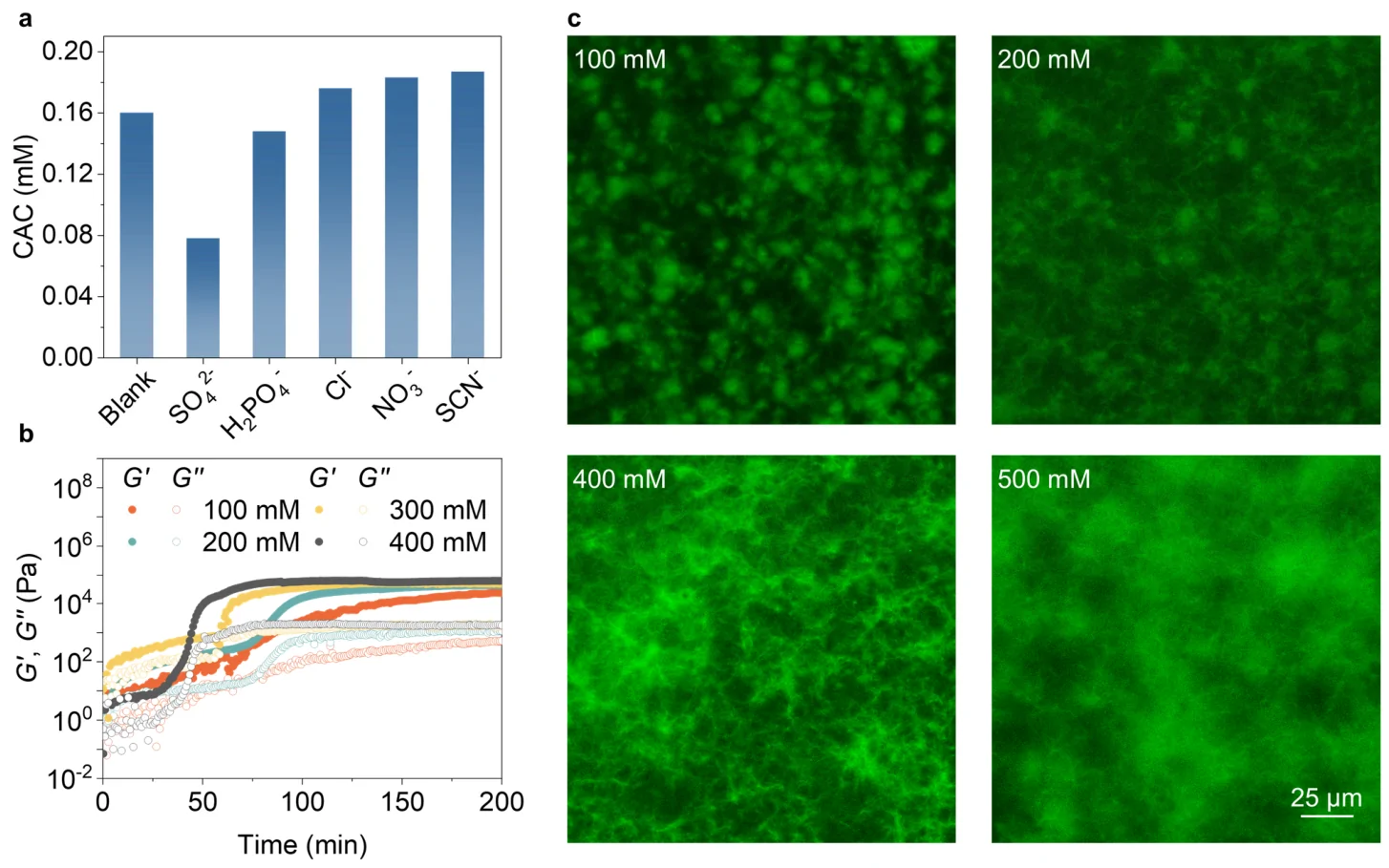
Mechanism of the effects of anions on the gelation behavior. (**a**) The model reaction between NBDH and A for kinetic measurement; (**b**) evolution of G′ and G″ of the samples with different [SO_4_^2−^] over time; and (**c**) confocal microscopy images showing the hydrogel networks formed with different [SO_4_^2−^].

Therefore, it is very likely that these anions influence the gelation behavior by modulating the intermolecular interactions among the gelators, which follows the mechanism of the Hofmeister effect. To test this, we measured the CAC of HA_3_ in the presence of different anions at 500 mM (see Supporting Information). Compared with the CAC of 0.159 mM without additional anions, the addition of kosmotropic anions (SO_4_^2−^ and H_2_PO_4_^−^) decreased the CAC to 0.078 mM and 0.148 mM, respectively (Figure 5a and Figure S7), indicating the strengthened intermolecular interactions. In contrast, the CAC was higher than 0.176 mM in the presence of chaotropic anions, which is significantly higher than in the case of kosmotropic anions, suggesting a strong propensity of the gelator to dissolve rather than to aggregate. These results are consistent with the Hofmeister effect and support the above hypothesis.

After demonstrating the influences of the Hofmeister effect on the self-assembly of the supramolecular hydrogels, we next systematically explored the influences of anion concentration on the gelation process and the hydrogel properties. Taking SO_4_^2−^ as an example, the maximum *G’* increased progressively with increasing anion concentration. When the concentration was increased from 100 to 500 mM, the *G’* rose from 22 to 117.4 kPa, while the gelation time decreased from 152 to 58 min (Figure 3b and Figure 5b). Confocal microscopy images revealed that as [SO_4_^2−^] increased from 100 to 500 mM, the density of the fibrous network gradually increased (Figure 5c), which can originate from the increased self-assembly capacity.

**Figure 6:**
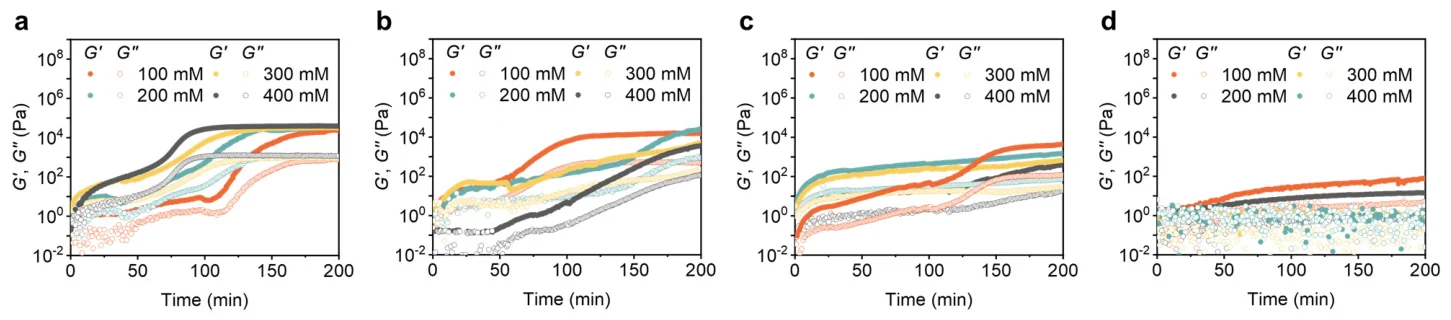
Evolution of *G’* and *G”* of the samples with different (**a**) [H_2_PO_4_^−^]; (**b**) [Cl^−^]; (**c**) [NO_3_^−^]; and (**d**) [SCN^−^] over time.

Similar results are obtained from the samples in the presence of H_2_PO_4_^−^ (Figure 6a). The maximum *G’* increased progressively with the [H_2_PO_4_^−^]. When the concentration was increased from 100 to 500 mM, the *G’* rose from 23.7 to 65.2 kPa, while the gelation time decreased from 172 to 61 min (Figure 3b, Figure 6a and Figure S8). Meanwhile, density variation of the hydrogel network against [SO_4_^2−^] is also observed in the case of H_2_PO_4_^−^ (Figure S9). In stark contrast, reversed effects on the rheological properties were observed in the case of chaotropic anions (Figure 6b–d and Figure S10). The maximum *G’* decreased progressively when the concentration was increased from 100 to 500 mM. Meanwhile, confocal microscopy images revealed that with the increase in concentration of chaotropic anions from 100 to 500 mM, the density of the fibrous network gradually decreased, leading to large clusters (Figure S11). Somehow, increasing the [Cl^−^] from 100 to 200 mM unexpectedly enhanced the maximum *G’* from 15.7 kPa to 26.1 kPa. This result is likely caused by the kinetic promotion of hydrazone formation by Cl^−^ anions, leading to a denser fibrous network (Figure 6b). Meanwhile, all hydrogel samples under gelation conditions exhibited typical gel-like behavior, with G’ dominating over G’’ across the entire frequency range. The strain sweep measurements revealed that the yield points showed no significant differences among samples under the same ionic conditions with different concentrations, indicating comparable network stability once the gels are fully formed. These results proved that the Hofmeister effect can serve as an effective approach to control the self-assembly of supramolecular hydrogels and their material properties.

## 3. Conclusions

In summary, on the basis of a hydrazone formation-mediated supramolecular gelation system, we have demonstrated that anions possess important effects on the self-assembly of supramolecular hydrogels following the Hofmeister series. The addition of kosmotropic anions (SO_4_^2−^, H_2_PO_4_^−^) can accelerate the self-assembly of gelators, giving rise to supramolecular hydrogels with denser fibrous networks and higher stiffness. In contrast, the addition of chaotropic anions (Cl^−^, NO_3_^−^, SCN^−^) effectively impedes the self-assembly process, leading to the formation of large and sparse fibrous clusters and failure of gelation. These effects are in good agreement with the Hofmeister effect, which is thought to influence the intermolecular interactions by changing the solvation structures of the solutes [35,48]. We have verified that, in contrast to chaotropic anions, the kosmotropic anions can indeed decrease the critical aggregation concentration of the gelator, suggesting their capability of accelerating the self-assembly of gelators. This work suggests that the Hofmeister effect could significantly influence the self-assembly and properties of supramolecular hydrogels, offering an alternative approach to make supramolecular hydrogels with desired structures and functions.

## 4. Materials and Methods

### 4.1. Materials and Instruments

All the commercial chemicals were purchased from Sigma Aldrich (St. Louis, MO, USA) and utilized without further purification. Precursors H, A, NBDH [54] and fluorescein-labeled aldehyde derivative (A-FL) [55] were synthesized according to the previously reported methods. UV-Vis absorption spectra were recorded using a quartz cuvette of 1.0 cm path length on a Shimadzu UV-1900i spectrophotometer (Suzhou, China) equipped with a Shimadzu TCC-100 cell holder for temperature control. The confocal laser scanning microscope (CLSM) images were collected from a Leica DMI8 confocal microscope (Wetzlar, Germany). The NMR spectra were recorded on a BRUKER ASCEND 400 (Billerica, MA, USA) operating at 400 MHz.

### 4.2. Preparation of Stock Solutions

Stock solutions of precursors H and A were prepared by dissolution in 10 mM phosphate buffer (pH 6.0) at the concentrations of 40 and 400 mM, respectively. Salts were dissolved in phosphate buffer (10 mM, pH 6.0) at a stock concentration of 3 M. The pH of all salt stock solutions was verified and, when required, adjusted to 6.0 using 0.125 mM NaOH. The minimal volume of NaOH solution employed for pH adjustment ensured negligible impact on the overall ionic strength of the solutions. The final pH of all the samples were confirmed to ensure that the pH was maintained at 6.0.

For critical aggregation concentration (CAC) determination, a 1.0 mM stock solution of Nile Red was prepared in DMSO. All experimental samples were subsequently derived through appropriate dilutions of these stock solutions using the respective solvents.

### 4.3. Kinetic Measurements on Hydrazone Formation Under Different Anionic Conditions

The influence of various anions on hydrazone bond formation kinetics was investigated through UV-Vis spectroscopic analysis (SHIMADZU UV-1900i) maintained at 25 °C. Reaction mixtures (300 μL) containing 0.045 mM NBDH, 2.5 mM A, and 100 mM sodium salts with distinct anions were prepared from pre-configured stock solutions. The concentrations of anions and reactants were lower than in the gelation experiment to meet the requirements of UV measurements. Following vortex mixing, samples were immediately transferred to quartz cuvettes with a 1 mm slit width, with time-dependent absorbance measurements recorded continuously at 480 nm (0.1 min intervals) to track the characteristic absorption of the hydrazone product. The relative reaction rates (*k*_rel._) were determined from the linear slopes of corresponding kinetic curves and normalized to the data from the control sample without added anions.

### 4.4. Measurements of Critical Aggregation Concentration (CAC)

The critical aggregation concentration (CAC) of gelator molecules HA_3_ in different sodium salt systems was determined using Nile red as a fluorescent probe. The detailed procedure was as follows: For each test, mixed solutions containing gradient concentrations of H (0.01–10 mM) and A (0.06–60 mM) solution were prepared from stock solutions. These were combined with sodium salts (500 mM) in centrifuge tubes containing 10 μM Nile red solution. The mixture was vortexed for 3 s to ensure homogeneity and then incubated in the dark at room temperature for 36 h to reach thermodynamic equilibrium. Emission spectra were acquired using a Cary Eclipse fluorescence spectrophotometer (Santa Clara, CA, USA) at an excitation wavelength of 550 nm, with both excitation and emission slit widths set to 10 nm and the PMT voltage maintained at 650 V. Fluorescence intensity was simultaneously monitored at 635 nm during spectral acquisition.

### 4.5. Rheological Tests

The mechanical properties of the hydrazone-based hydrogels were characterized by oscillatory rheological measurements on an Anton Paar Physica MCR 401 rheometer (Graz, Austria) equipped with a 25 mm parallel-plate geometry. The gap was fixed at 0.4 mm, and the plate temperature was maintained at 25 ± 0.2 °C. For time sweep measurements, the strain and frequency were set to 0.05% and 1.0 Hz, respectively. Frequency sweep measurements were conducted at a constant strain of 0.05% over the frequency range of 0.1–100 Hz, while strain sweep measurements were performed at a constant frequency of 1.0 Hz over a strain range of 0.01–100%. All samples contained 20 mM H and 120 mM A and were prepared by mixing with aqueous sodium salt solutions at concentrations ranging from 0 to 500 mM. Immediately after thorough mixing, 200 μL of the freshly prepared sample was carefully transferred onto the lower plate of the rheometer. The upper plate was then lowered slowly to the preset gap, and excess sample at the plate edge was gently removed using a spatula. To minimize solvent evaporation during the measurements, a water trap was employed throughout the test. Consecutive time sweep, frequency sweep, and strain sweep measurements were then carried out. The complete testing protocol was repeated for each sample to ensure data comparability and reproducibility.

### 4.6. Confocal Laser Scanning Microscopy (CLSM)

CLSM observations were performed using a LEICA TCS SP8 confocal laser scanning microscope (Wetzlar, Germany) equipped with a Leica microscope and a 63× immersion objective using an incident laser with a wavelength of 488 nm to excite the fluorescein probe. The pinhole was set to 1.0 airy unit during the measurement and the data were processed using Leica Application Suite X software (LAS_X_4.7.0). Each sample was prepared in cell culture plastics and was incubated at 25 °C overnight before the measurements.

## Data Availability

The data presented in this study are openly available in the article. The data supporting this article have been included as part of the Supplementary Information (SI). Supplementary Information is available.

## Supplementary Materials

The following supporting information can be downloaded at: https://www.mdpi.com/article/10.3390/gels12080693/s1, Figure S1: ^1^H-NMR spectra of H (DMSO-*d*_6_).; Figure S2: ^1^H-NMR spectra of A (CDCl_3_); Figure S3: ^1^H-NMR spectra of NBDH (D_2_O); Figure S4: Transmittance of the hydrogels determined by UV-Vis spectra at 600 nm; Figure S5: Molecular structure of aldehyde-derived fluorescein A-FL; Figure S6: Dependence of *G^′^* and *G^″^* of the hydrogels on frequency and strain with different anions. [Anions] = 500 mM; Figure S7: The absorbance intensity at 635 nm of the solutions with varying [HA_3_] and different anions. [Nile Red] = 10 μM, [Anions] = 500 mM. The inflection concentration at which the intensity abruptly increases is determined as the corresponding CAC of HA_3_; Figure S8: The dependence of the hydrogel storage modulus (G^′^) on frequency and strain with different (a) [SO_4_^2−^] and (b) [H_2_PO_4_^−^]. The frequency and strain sweeps were performed immediately after completion of the corresponding time sweep measurement, without removing the sample from the rheometer. Sample: [H] = 20 mM, [H]:[A] = 1:6 in 10 mM phosphate buffer (pH 6.0); Figure S9: Confocal microscopy images showing the hydrogel networks formed with different [H_2_PO_4_^−^]. Samples: [H] = 20 mM, [A] = 120 mM and [A-FL] = 30 μM in 10 mM phosphate buffer (pH = 6.0); Figure S10: The dependence of the hydrogel storage modulus (*G^′^*) on frequency and strain with different (a) [Cl^−^], (b) [NO_3_^−^], and (c) [SCN^−^]. The frequency and strain sweeps were performed immediately after completion of the corresponding time sweep measurement, without removing the sample from the rheometer. Sample: [H] = 20 mM, [H]:[A] = 1:6 in 10 mM phosphate buffer (pH 6.0); Figure S11: Confocal microscopy images showing the hydrogel networks formed with different a) [Cl^−^], b) [NO_3_^−^], and [SCN^−^]. Samples with 400 and 500 mM SCN^−^ are not available (N.A.). Samples: [H] = 20 mM, [A] = 120 mM and [A-FL] = 30 μM in 10 mM phosphate buffer (pH = 6.0).
