# Supplementary material for: Control over the Self-Assembly of Supramolecular Hydrogels Using Hofmeister Effect
Source: Gels. 2026 Aug 4;12(8):693. doi: 10.3390/gels12080693 (PMC13512813; doi:10.3390/gels12080693)
Supplement: Supplementary file 1 [file gels-12-00693-s001.zip › gels-4466858-supplementary.pdf]

Supporting Information

Control Over the Self-Assembly of Supramolecular Hydrogels Using Hofmeister Effect

Lai Wei,[a, #] Qi Gao,[a, #] Hongwang Tang,[a] Xuhong Guo,[a] Yiming Wang*[a,b]

[a]State Key Laboratory of Chemical Engineering, East China University of Science and Technology, Shanghai 200237, China.

[b]Shanghai Key Laboratory for Intelligent Sensing and Detection Technology, East China University of Science and Technology, Shanghai 20037, China.

*Email: yimingwang@ecust.edu.cn

# These authors contribute equally to this work

Table of Contents

Figure S1. ^{1}H-NMR spectra of H. S1

Figure S2. ^{1}H-NMR spectra of A. S1

Figure S3. ^{1}H-NMR spectra of NBDH. S2

Figure S4. Transmittance of the hydrogels determined by UV-Vis spectra at 600 nm. S2

Figure S5. Molecular structure of aldehyde-derived fluorescein A-FL. S2

Figure S6. Dependence of G' and G" of the hydrogels on frequency and strain with different anions. S3

Figure S7. CAC of HA_3 in the presence of different anions. S3

Figure S8. Rheological tests of kosmotropic anions. S4

Figure S9. Confocal microscopy images of samples under different [$H_2PO_4^-$] S4

Figure S10. Rheological tests of chaotropic anions. S5

Figure S11. Confocal microscopy images of samples under different [kosmotropic anions]. S6

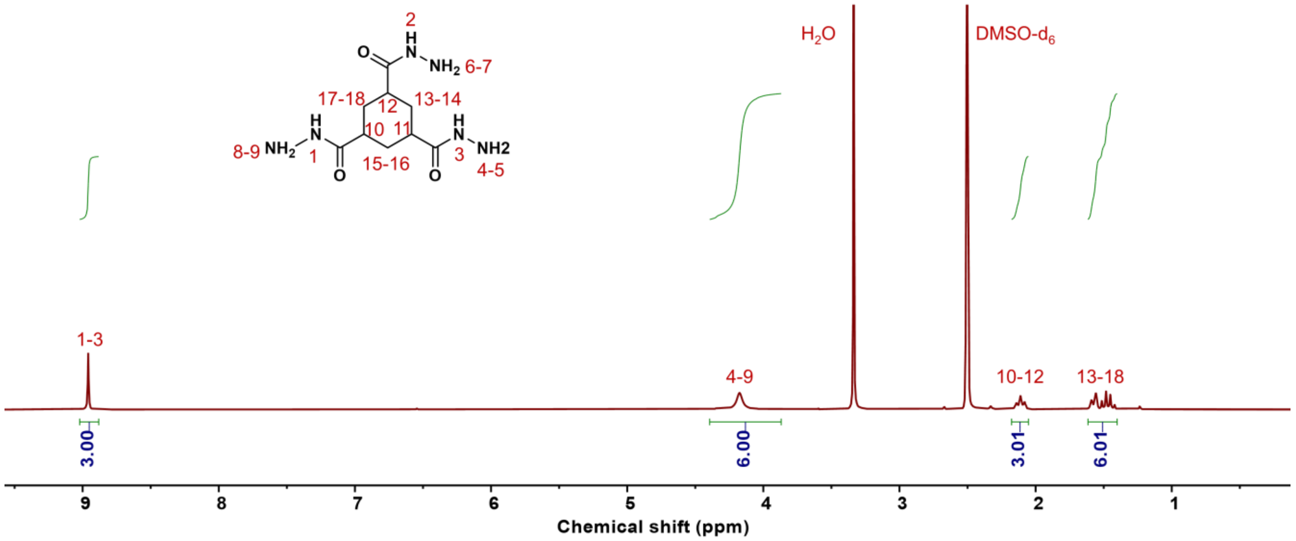

Figure S1. ^{1}H-NMR spectra of H (DMSO-d_6).

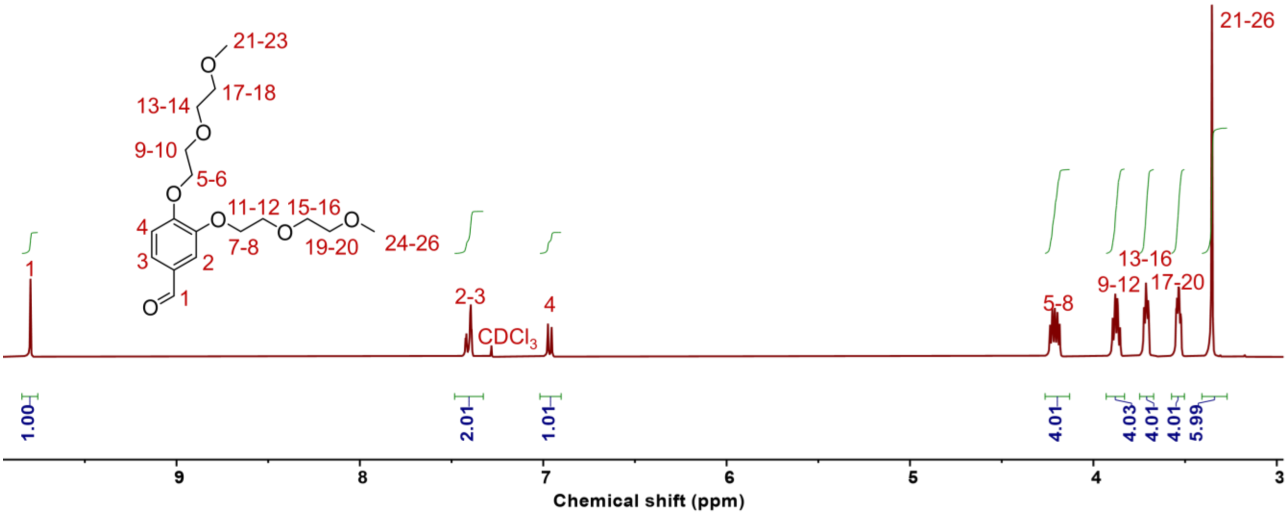

Figure S2. ^{1}H-NMR spectra of A ($CDCl_3$).

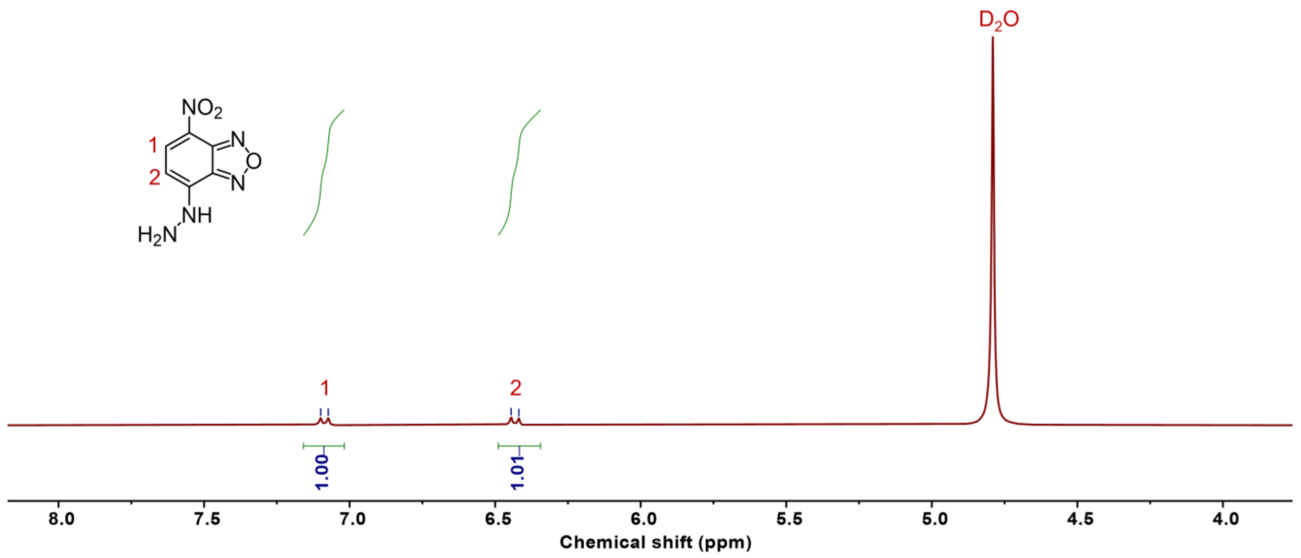

Figure S3. ^{1}H-NMR spectra of NBDH (D_2O).

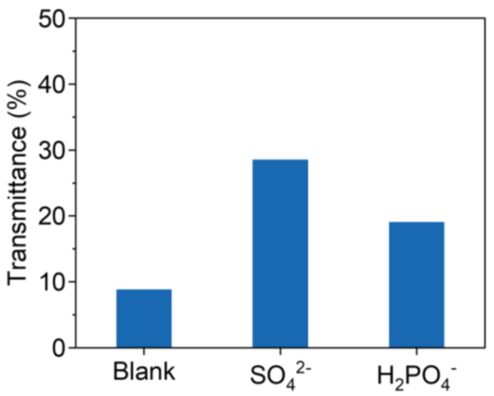

Figure S4. Transmittance of the hydrogels determined by UV-Vis spectra at 600 nm.

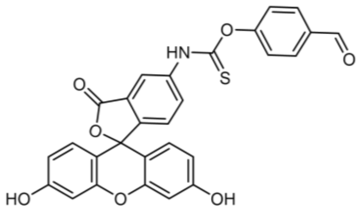

Figure S5. Molecular structure of aldehyde-derived fluorescein A-FL.

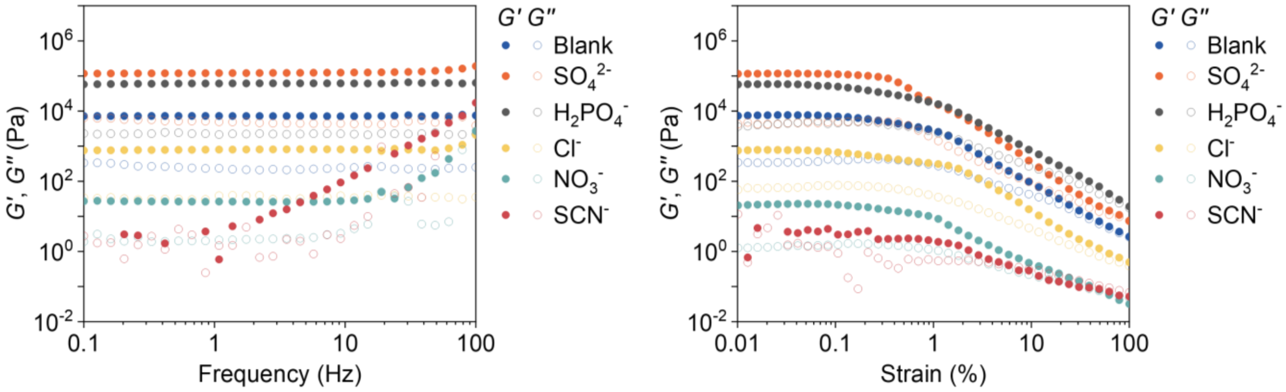

Figure S6. Dependence of *G'* and *G''* of the hydrogels on frequency and strain with different anions. [Anions] = 500 mM.

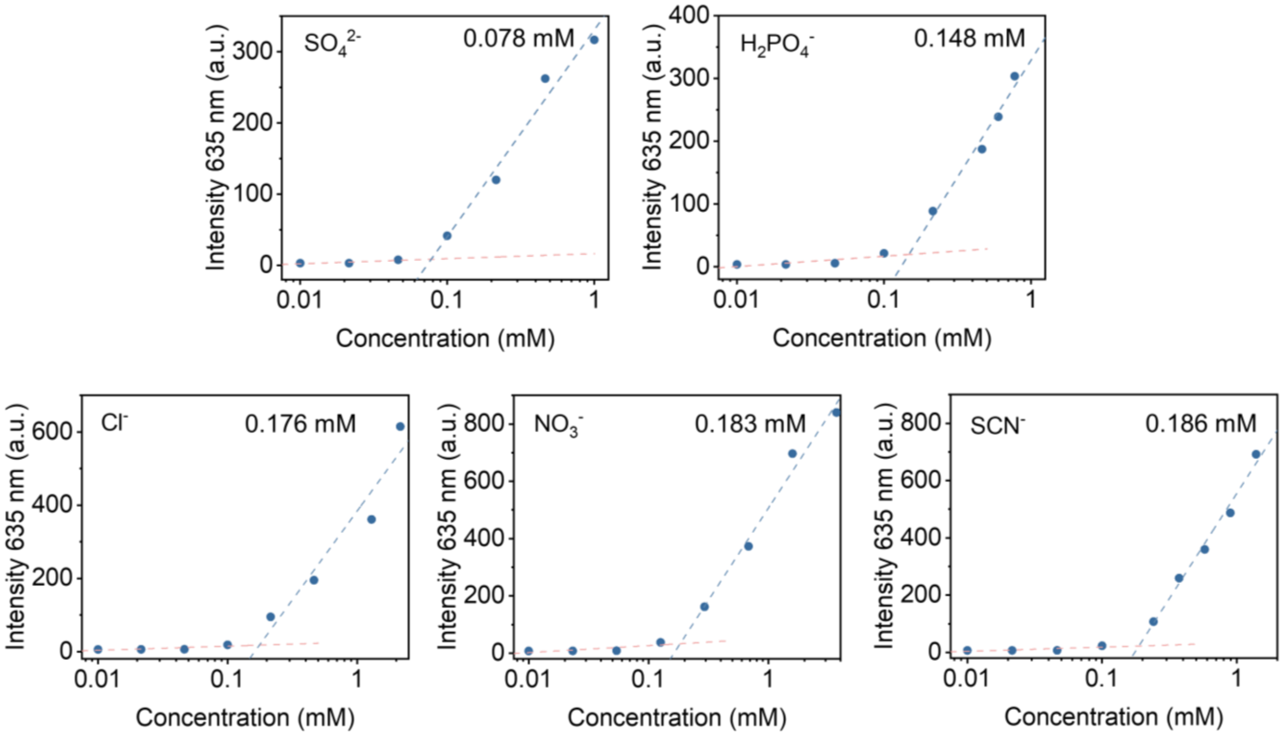

Figure S7. The absorbance intensity at 635 nm of the solutions with varying [HA3] and different anions. [Nile Red] = 10 µM, [Anions] = 500 mM. The inflection concentration at which the intensity abruptly increases is determined as the corresponding CAC of HA3.

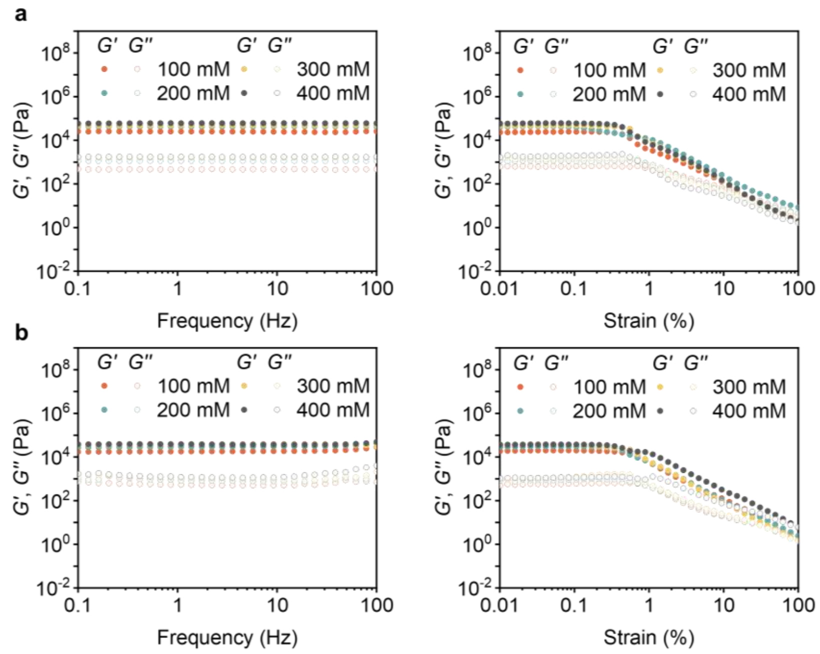

Figure S8. The dependence of the hydrogel storage modulus (G') on frequency and strain with different (a) [SO_4^{2-}] and (b) [$H_2PO_4^-$]. The frequency and strain sweeps were performed immediately after completion of the corresponding time sweep measurement, without removing the sample from the rheometer. Sample: [H] = 20 mM, [H]:[A] = 1:6 in 10 mM phosphate buffer (pH 6.0).

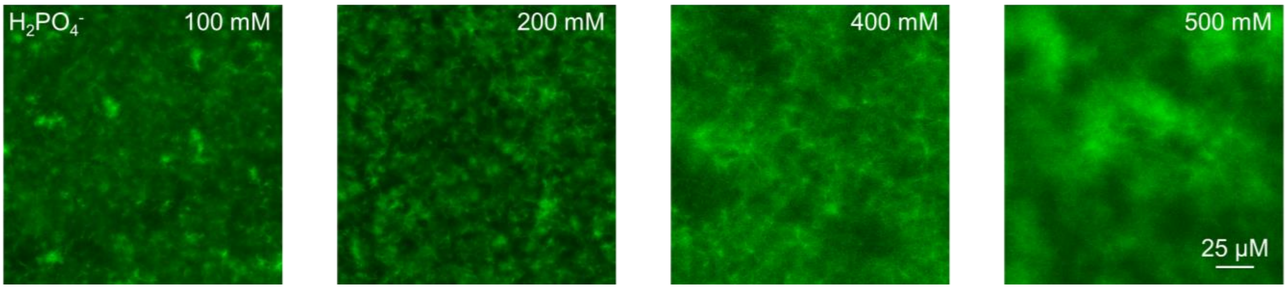

Figure S9. Confocal microscopy images showing the hydrogel networks formed with different [$H_2PO_4^-$]. Samples: [H] = 20 mM, [A] = 120 mM and [A-FL] = 30 μM in 10 mM phosphate buffer (pH = 6.0).

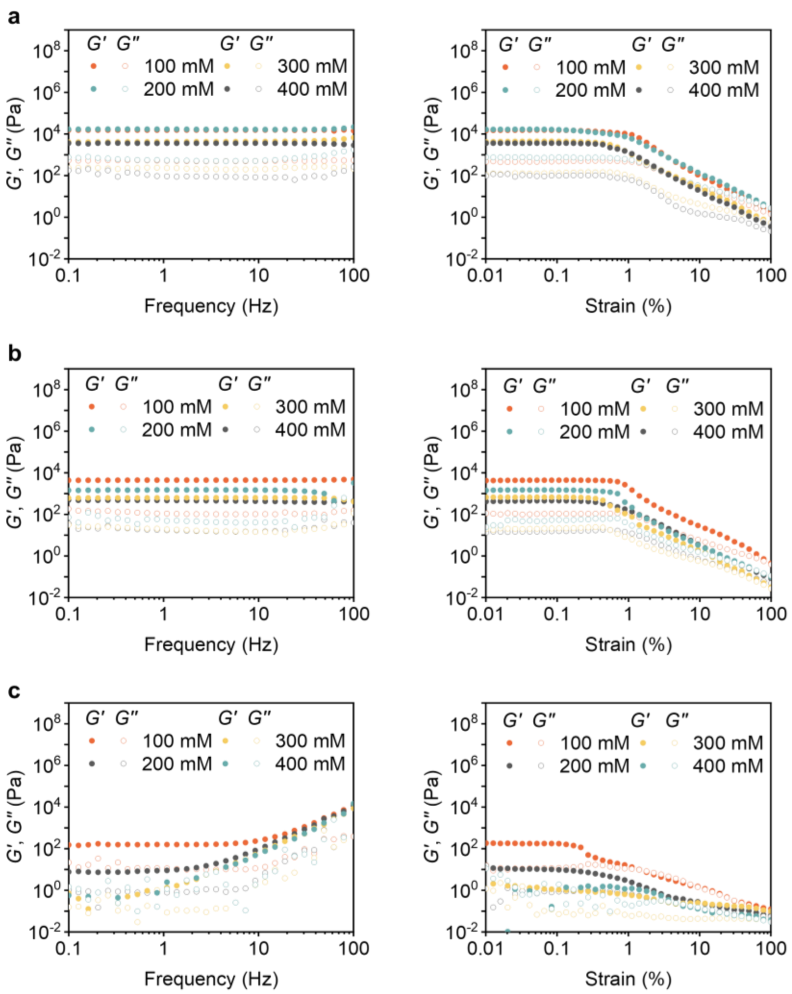

Figure S10. The dependence of the hydrogel storage modulus (*G′*) on frequency and strain with different (a) [Cl^-], (b) [NO_3^-], and (c) [SCN^-]. The frequency and strain sweeps were performed immediately after completion of the corresponding time sweep measurement, without removing the sample from the rheometer. Sample: [H] = 20 mM, [H]:[A] = 1:6 in 10 mM phosphate buffer (pH 6.0).

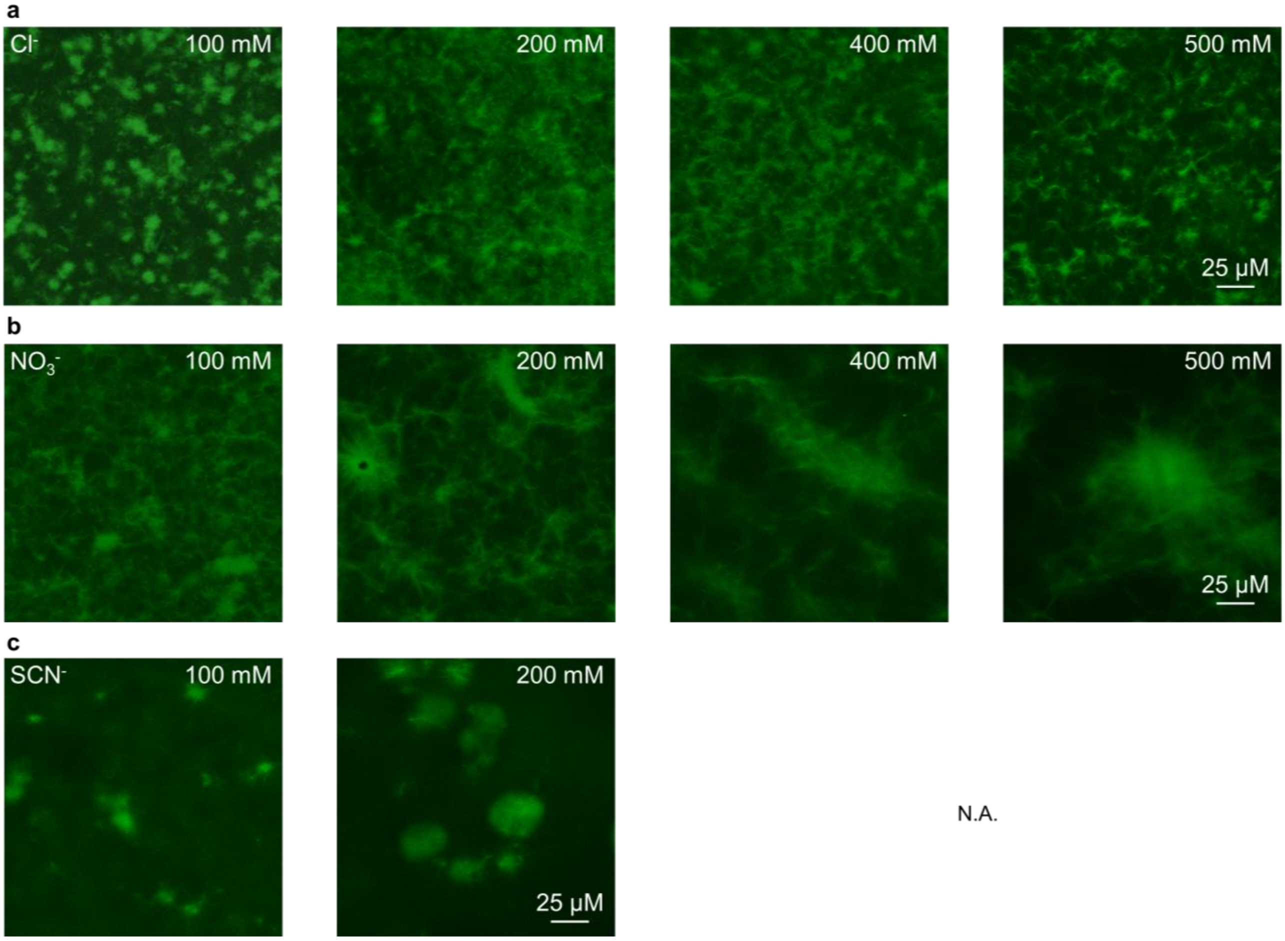

Figure S11. Confocal microscopy images showing the hydrogel networks formed with different a) [Cl^-], b) [NO_3^-], and [SCN^-]. Samples with 400 and 500 mM SCN^- are not available (N.A.). Samples: [H] = 20 mM, [A] = 120 mM and [A-FL] = 30 μM in 10 mM phosphate buffer (pH = 6.0).
